# Global Status Report for the Verification of Measles and Rubella Elimination, 2022

**DOI:** 10.3390/vaccines12080947

**Published:** 2024-08-22

**Authors:** Patrick O’Connor, Balcha Masresha, Desirée Pastor, Nasrin Musa, José Hagan, Sudhir Khanal, Chung-Won Lee, Natasha Crowcroft

**Affiliations:** 1 World Health Organization, Headquarters, 1211 Geneva, Switzerland; crowcroftn@who.int; 2 World Health Organization, Regional Office for Africa, Brazzaville P.O. Box 06, Congo; masreshab@who.int; 3 World Health Organization, Regional Office for Americas, Washington, DC 20037, USA; dpastor@paho.org; 4 World Health Organization, Regional Office for the Eastern Mediterranean, Cairo 11371, Egypt; musan@who.int; 5 World Health Organization, Regional Office for Europe, 2100 Copenhagen, Denmark; haganj@who.int; 6 World Health Organization, Regional Office for South-East Asia, New Delhi 110002, India; khanals@who.int; 7 World Health Organization, Regional Office for Western Pacific, Manila 1000, Philippines; leech@who.int

**Keywords:** measles, rubella, elimination, verification

## Abstract

Since the World Health Assembly (WHA) in 2012 endorsed the Global Vaccine Action Plan (GVAP), which included regional measles and rubella elimination goals by 2020, global progress towards verification of measles and rubella elimination has been incremental. Even though the 2020 elimination goals were not achieved, commitment towards achieving measles and rubella elimination has been firmly established in the Immunization Agenda 2030 (IA2030) and the Measles and Rubella Strategic Framework (MRSF) 2021–2030. In 2023, the six Regional Verification Commissions for measles and rubella elimination (RVCs) reviewed data as of 31 December 2022 and confirmed that 82 (42%) Member States have been verified for measles elimination, and 98 (51%) Member States have been verified for rubella elimination. The six RVCs are composed of independent public health and immunization experts who are well-placed to support accelerating measles and rubella elimination. RVCs should be leveraged not only to review elimination documents but also to advocate for and champion public health programming that supports measles and rubella activities. The verification of elimination process is one of many tools that should be deployed to reinforce and accelerate efforts towards achieving a world free of measles and rubella.

## 1. Introduction: History, Background, and Context

There has been considerable progress towards achieving measles and rubella elimination and reducing the burden caused by measles and rubella since the World Health Organization (WHO) convened an expert advisory panel in 2010, which concluded measles can and should be eradicated [1]. In November 2010, the WHO Strategic Advisory Group of Experts on Immunization (SAGE) endorsed the conclusion of the expert advisory panel; and in 2012, the World Health Assembly (WHA) endorsed the Global Vaccine Action Plan (GVAP), which included measles and rubella elimination goals by 2020 [2,3]. The ambitious regional elimination goals of GVAP were not attained and the Region of the Americas (AMR) was the only WHO region to achieve and maintain its regional rubella elimination goal [4,5]. However, the global, regional, and national commitment to achieve these goals continues through the implementation of the Immunization Agenda 2030 (IA2030) and the Measles and Rubella Strategic Framework (MRSF) with the ultimate goal of a world free of measles and rubella [6,7]. Immunization activities aimed at achieving and maintaining measles and rubella elimination will advance the following: (1) the IA2030 goal to prevent 50 million deaths by 2030; modeling estimates that measles vaccine will account for 37% of deaths averted between 2021–2030; and, (2) the MRSF goal to complete the introduction of rubella vaccines into routine immunization schedules and prevent the estimated 32,000 cases of congenital rubella syndrome, which is the leading cause of vaccine-preventable birth defects [8,9].

As of April 2024, all six WHO Regions have Regional Committee resolutions with endorsement by Member States and commitment to achieve measles elimination [10,11,12,13,14,15]. Because of the range in immunization coverage and disease burden across Member States, the target dates for regional measles elimination goals vary. In addition, five of the six WHO Regions have Regional Committee resolutions with endorsement by Member States and commitment to achieve rubella elimination. The sixth region, WHO Eastern Mediterranean Region (EMR), the final region, is in the process of developing a regional rubella elimination goal that will be presented at a future Regional Committee for consideration and endorsement. Despite not having a rubella elimination goal yet, several countries in the EMR have already been verified for the elimination of rubella. This report provides a global update on the current situation on measles and rubella elimination, challenges, and potentials for acceleration.

## 2. Materials and Methods

The verification statuses of measles and rubella elimination for WHO Member States were reviewed from the meeting reports of the six regional verification commissions (RVCs) that were conducted in 2023. The RVCs reviewed immunization and surveillance data submitted by National Verification Committees (NVCs) as of 31 December 2022. Relevant measles and rubella elimination documents and guidance were also reviewed to provide context and historical perspective. Guidance for the verification of measles and rubella elimination including lines of evidence, suggested composition of RVCs and NVCs, and documentation is outlined in the Weekly Epidemiological Record (WER) report of October 2018 [16].

## 3. Results: Measles and Rubella Elimination Progress 2022

The regional verification commissions (RVCs) are responsible for reviewing the national reports prepared and submitted by the National Verification Committees (NVCs) and provide an assessment of the elimination status for each Member State. The Weekly Epidemiological Record (WER) report of October 2018 provides the most recent guidance on the agreed process for evaluating measles and rubella elimination status [16]. Throughout 2023, all six of the RVCs reviewed the elimination status of the 194 WHO Member States and 11 territories for data ending 31 December 2022. During the COVID-19 pandemic, many of the RVCs held virtual meetings; 2023 was the first year that all the RVCs held in-person meetings:

- the African Regional Verification Commission (AF-RVC) met in May 2023 [17];
- the South-East Asian Regional Verification Commission (SEA-RVC) in June 2023 [18];
- the Western Pacific Regional Verification Commission (WP-RVC) in September 2023 [19];
- the European Regional Verification Commission (EU-RVC) in September 2023 [20];
- the Region of the Americas—measles, rubella, and congenital rubella syndrome post-elimination Regional Monitoring and Re-Verification Commission in November 2023 [21];
- and the Eastern Mediterranean Regional Verification Commission (EM-RVC) in December 2023 [22].

While some of the regions have developed elimination classifications and categories to guide national immunization programmes and provide actionable feedback to Member States, the guidance outlined in the 2018 WER report has four elimination categories: (1) endemic—continuous transmission of measles and/or rubella that persists for greater than or equal to 12 months in any defined geographic area and no previous verification of elimination; (2) eliminated—absence of endemic transmission or a continuous period of greater than or equal to 12 months in the presence of high-quality surveillance systems; (3) verified—no endemic virus transmission for a continuous period of greater than or equal to 36 months in the presence of a high-quality surveillance system and confirmed by the RVC; and (4) re-established endemic transmission post-verification—the presence of a chain of transmission that continues uninterrupted for greater than or equal to 12 months in a defined geographic area (region or country) after previous verification of elimination [15]. Classifications provided in the 2022 regional verification reports have been aligned with the WER 2018 guidance.

Table 1 summarizes the current measles and rubella elimination status and is based on data as of 31 December 2022. For measles elimination, the results are as follows: 82 (42%) Member States were classified as verified, 21 (11%) Member States were classified as eliminated, 85 (43%) Member States were classified as endemic, 5 (3%) Member States were classified as re-established endemic transmission post-verification, and 1 (1%) Member State did not submit a report for review. For rubella elimination, the results were as follows: 98 (50%) Member States were classified as verified, 13 (7%) Member States were classified as eliminated; 82 (42%) Member States were classified as endemic; no (0%) Member States were classified as re-established endemic transmission post-verification; and 1 (1%) Member State did not submit a report for review.

**Figure 1:**
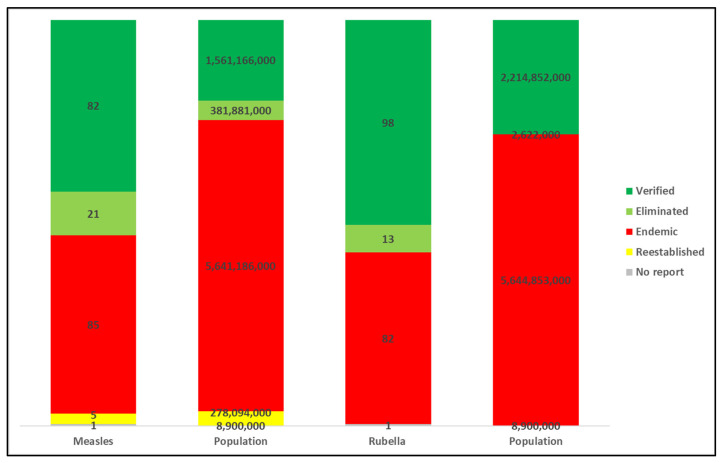
Measles and rubella elimination categories * by number of WHO Member States and total population ^†^, 2022. Abbreviation: WHO = World Health Organization. [* Categories for classifying the elimination status of countries and territories and definitions are derived from the Weekly Epidemiological Record (WER) 12 October 2018 (93): 544–552 [16]. Guidance for evaluating progress towards elimination of measles and rubella. https://iris.who.int/bitstream/handle/10665/275394/WER9341-544-552.pdf?sequence=1&isAllowed=y (accessed on 30 April 2024). ^†^ United Nations, Department of Economic and Social Affairs, Population Division (2022). World Population Prospect 2022, Online Edition. World Population Prospects—Population Division—United Nations].

Analyzing the measles and rubella elimination classifications by total population provides an additional lens on global progress and the challenges particularly for large countries to achieve and maintain measles and rubella elimination [Figure 1]. For measles elimination, the numbers were as follows: 1,561,166,000 (20%) persons reside in Member States classified as verified; 381,881,000 (5%) persons reside in Member States classified as eliminated; 5,641,186,000 (72%) persons reside in Member States classified as endemic; 278,094,000 (4%) persons reside in Member States classified as re-established endemic transmission post-verification; and 8,900,000 (<1%) persons reside in a Member State that did not provide a report. For rubella elimination, the numbers were as follows: 2,214,852,000 (28%) persons reside in Member States classified as verified; 2,622,000 (<1%) persons reside in Member States classified as eliminated; 5,644,853,000 individuals reside in Member States classified as endemic (71%); no (0%) persons reside in Member States classified as re-established endemic transmission post-verification; and 8,900,000 (<1%) persons reside in a Member State that did not provide a report. Table 2 and Table 3 summarize the 2022 measles and rubella elimination status by WHO Region and elimination status, respectively. Table 4 summarizes the 2022 measles and rubella elimination status by WHO Member State and national population. Currently, the only regional elimination goal that has been achieved and maintained is rubella elimination in the WHO AMR, which has maintained this status since 2015.

## 4. Discussion: Accelerating Verification of Measles and Rubella Elimination

While the regional measles and rubella elimination goals outlined in the GVAP and endorsed by the WHA were not fully achieved by 2020 [4], there has been progress toward achieving and maintaining elimination: 42% of Member States have been verified for measles elimination and 51% of Member States have been verified for rubella elimination. The challenges towards achieving measles and rubella elimination can be categorized into two main groups: (1) interrupting endemic transmission supported by a well-performing measles and rubella surveillance system, and (2) documenting elimination for verification.

First, interrupting endemic transmission of measles and rubella requires high, uniform, and equitable immunization coverage. Ensuring that all Member States have routine immunization programs with two doses of a measles and rubella vaccine is critical. As of the beginning of 2024, there are 19 Member States that need to fully introduce a rubella vaccine and four Member States that need to introduce a second measles vaccine into their national immunization program [23]. Completing this work is critical to establishing equitable conditions, ensuring that all eligible individuals have access to measles and rubella vaccines, and achieving high immunity to these viruses. Reaching 95% coverage with two routine doses of measles and rubella vaccines is a global challenge and when that target is not achieved in a single year or over many years, immunization gaps can emerge, resulting in an increasing risk of measles outbreaks.

Developing timely, regular opportunities to catch up and deliver doses missed by the routine program is critical to having population immunity high enough to stop endemic transmission and prevent the morbidity and mortality associated with measles and rubella infections. These opportunities may take different forms in different places: enhanced routine immunization sessions with record or immunization card review, call-back services, and defaulter tracing; mobile and outreach immunization services to communities with limited access; targeted immunization campaigns for specific geographic locations, age groups, or occupations; and large-scale, non-selective nationwide campaigns. Initiatives like the *Big Catch-up* and strategies to expand eligibility have been developed to support the post-COVID-19 pandemic recovery of immunization services and ensure that missed routine doses are received [24,25]. In addition to the doses provided by routine immunization programs and regular catch-up opportunities, robust outbreak preparedness and response will be necessary to interrupt chains of transmission and rapidly boost population immunity. Immunization activities need to be complemented by well-performing, sensitive, laboratory-supported measles and rubella surveillance systems.

Secondly, adequately documenting elimination for verification can be challenging and requires an in-depth analysis of current and historical data to develop a national report that follows the five lines of evidence for verifying elimination: (1) detailed description of the current and past epidemiology of measles, rubella, and congenital rubella syndrome (CRS); (2) analysis of molecular epidemiology to document viral transmission patterns and the duration of circulation of viruses of specific lineages; (3) quality of surveillance and monitoring systems for measles, rubella, and CRS; (4) population immunity presented as a birth cohort analysis, with subanalysis on adults, underserved communities, migrants, and refugee groups; and, (5) accountability, ownership, and political commitment [15].

The national-level report needs to be submitted to the RVC by an established, functioning NVC with a supporting secretariat. The initial documentation and subsequent analysis to demonstrate interruption of virus transmission can be time-consuming and has in many Member States been supported by global and regional measles and rubella partners. Even countries that may be far from achieving measles and rubella elimination can benefit from the process of preparing verification documents and having the NVC submit a report to the RVC for review. It is an opportunity for an annual review of the national immunization program, surveillance system, and outbreak response by a group of independent public health and immunization experts and should be leveraged as an important tool for accelerating elimination by national public health programs. Additionally, there are Member States with well-performing immunization programs, robust laboratory-supported vaccine-preventable disease surveillance systems, and rapid outbreak response mechanisms that have probably already interrupted endemic transmission but are missing an NVC and/or a report outlining progress. Additional technical support and advocacy may be needed to assist Member States in completing this required documentation.

The achievement in the Region of the Americas (AMR) of regional rubella elimination in 2015 and measles elimination in 2016 demonstrates that the tools for achieving elimination exist. While the use of innovations and new technologies such as measles–rubella rapid diagnostic tests and measle–rubella patch vaccines may help with accelerating progress, efforts to achieve and maintain the current elimination goals should not be delayed. The Region of the Americas also showed that maintaining regional measles elimination is difficult. Unfortunately, importations and ongoing chains of transmission ultimately resulted in the loss of the regional measles elimination status in 2018. It is important to recognize that measles and rubella elimination is a dynamic process [26]. Member States might find it challenging to interrupt endemic transmission for 12 months, or might achieve elimination and interrupt transmission for greater than 12 months but are unable to maintain it for 36 months to be verified, or might interrupt endemic transmission and are verified but re-establish transmission due to importations with transmission that lasts more than 12 months. The verification of measles and rubella elimination should not be seen as something to be achieved and forgotten, but as an ongoing process that requires high-level political and technical engagement and commitment.

The re-establishment of endemic transmission illustrates the need for a well-crafted national post-verification sustainability plan. While the WHO Regions and Member States are at different points on the pathway or stages towards measles and rubella elimination, maintaining focus on the ultimate goals of achieving a world free of measles and rubella should guide our current efforts towards improving routine immunization coverage, introducing a rubella vaccine and a second opportunity for a measles vaccine, planning regular supplementary activities to fill immunity gaps, and rapidly responding to outbreaks. The RVCs and NVCs are composed of independent public health and immunization experts who are well-placed to support accelerating measles and rubella elimination. RVCs and NVCs should be leveraged not only to develop and review elimination documents but also to advocate for and champion public health programming that supports all measles and rubella activities. The verification of elimination process is one of many tools that should be deployed to reinforce the efforts towards achieving a world free of measles and rubella.

## Figures and Tables

**Table 1:** Summary of WHO Member States’ measles and rubella elimination status by elimination categories and total populations, 2022.

Category *	Measles Elimination Category (%)	Total Population 2021 (Thousands) ^†^ (%)	Rubella Elimination Category (%)	Total Population 2021 (Thousands) ^†^ (%)
Verified	82	1,561,166	98	2,214,852
	(42%)	(20%)	(50%)	(28%)
Eliminated	21	381,881	13	2622
	(11%)	(5%)	(7%)	(<1%)
Endemic	85	5,641,186	82	5,644,853
	(43%)	(71%)	(42%)	(71%)
Re-established endemic transmission post-verification	5	278,094	0	0
	(3%)	(3%)	(0%)	(0%)
No report	1	8900	1	8900
	(1%)	(<1%)	(1%)	(<1%)
Total	194	7,871,227	194	7,871,227

Abbreviation: WHO = World Health Organization. [* Categories for classifying the elimination status of countries and territories and definitions are derived from the Weekly Epidemiological Record (WER) 12 October 2018 (93): 544–552 [16]. Guidance for evaluating progress towards elimination of measles and rubella. https://iris.who.int/bitstream/handle/10665/275394/WER9341-544-552.pdf?sequence=1&isAllowed=y (accessed on 30 April 2024). ^†^ United Nations, Department of Economic and Social Affairs, Population Division (2022). World Population Prospect 2022, Online Edition. World Population Prospects—Population Division—United Nations].

**Table 2:** Summary of measles elimination status by WHO Region and elimination categories, 2022.

Category *	WHO Region					
	African Region (AFR)	Region of the Americas (AMR)	Eastern Mediterranean Region (EMR)	European Region (EUR)	South-East Asia Region (SEAR)	Western Pacific Region (WPR)
Verified	0	34	4	33	5	6
Eliminated	0	0	0	8	0	13
Endemic	47	0	17	9	6	6
Re-established endemic transmission post-verification	0	1	0	2	0	2
No report	0	0	0	1	0	0
Total	47	35	21	53	11	27

Abbreviation: WHO = World Health Organization. [* Categories for classifying the elimination status of countries and territories and definitions are derived from the Weekly Epidemiological Record (WER) 12 October 2018 (93): 544–552 [16]. Guidance for evaluating progress towards elimination of measles and rubella. https://iris.who.int/bitstream/handle/10665/275394/WER9341-544-552.pdf?sequence=1&isAllowed=y] (accessed on 30 April 2024).

**Table 3:** Summary of rubella elimination status by WHO Region and elimination categories, 2022.

Category *	WHO Region					
	African Region (AFR)	Region of the Americas (AMR)	Eastern Mediterranean Region (EMR)	European Region (EUR)	South-East Asia Region (SEAR)	Western Pacific Region (WPR)
Verified	0	35	4	49	5	5
Eliminated	0	0	0	0	0	13
Endemic	47	0	17	3	6	9
Re-established endemic transmission post-verification	0	0	0	0	0	0
No report	0	0	0	1	0	0
Total	47	35	21	53	11	27

Abbreviation: WHO = World Health Organization. [* Categories for classifying the elimination status of countries and territories and definitions are derived from the Weekly Epidemiological Record (WER) 12 October 2018 (93): 544–552 [16]. Guidance for evaluating progress towards elimination of measles and rubella. https://iris.who.int/bitstream/handle/10665/275394/WER9341-544-552.pdf?sequence=1&isAllowed=y (accessed on 30 April 2024)].

**Table 4:** Measles and rubella elimination status of WHO Member State by elimination categories and total population, 2022.

WHO Member States	WHO Region	Measles Elimination Category 2022 *	Rubella Elimination Category 2022 *	Total Population 2021 (Thousands) ^†^
Afghanistan	EMR			40,099
Albania	EUR			2855
Algeria	AFR			44,178
Andorra	EUR			79
Angola	AFR			34,504
Antigua and Barbuda	AMR			93
Argentina	AMR			45,277
Armenia	EUR			2791
Australia	WPR			25,921
Austria	EUR			8922
Azerbaijan	EUR			10,313
Bahamas, The	AMR			408
Bahrain	EMR			1463
Bangladesh	SEAR			169,356
Barbados	AMR			281
Belarus	EUR			9578
Belgium	EUR			11,611
Belize	AMR			400
Benin	AFR			12,997
Bhutan	SEAR			777
Bolivia, Plurinational State of	AMR			12,079
Bosnia and Herzegovina	EUR			3271
Botswana	AFR			2588
Brazil	AMR			214,326
Brunei Darussalam	WPR			445
Bulgaria	EUR			6886
Burkina Faso	AFR			22,101
Burundi	AFR			12,551
Cabo Verde	AFR			588
Cambodia	WPR			16,589
Cameroon	AFR			27,199
Canada	AMR			38,155
Central African Republic	AFR			5457
Chad	AFR			17,180
Chile	AMR			19,493
China	WPR			1,425,893
Colombia	AMR			51,517
Comoros	AFR			822
Congo, Republic of	AFR			5836
Cook Islands	WPR			17
Costa Rica	AMR			5154
Côte d’Ivoire	AFR			27,478
Croatia	EUR			4060
Cuba	AMR			11,256
Cyprus	EUR			1244
Czechia	EUR			10,511
Democratic People’s Republic of Korea	SEAR			25,972
Democratic Republic of the Congo	AFR			95,894
Denmark	EUR			5854
Djibouti	EMR			1106
Dominica	AMR			72
Dominica Republic	AMR			11,118
Ecuador	AMR			17,798
Egypt, Arab Republic	EMR			109,262
El Salvador	AMR			6314
Equatoria Guinea	AFR			1634
Eritrea	AFR			3620
Estonia	EUR			1329
Eswatini (Swaziland)	AFR			1192
Ethiopia	AFR			120,283
Fiji	WPR			925
Finland	EUR			5536
France	EUR			64,531
Gabon	AFR			2341
Gambia, The	AFR			2640
Georgia	EUR			3758
Germany	EUR			83,409
Ghana	AFR			32,833
Greece	EUR			10,445
Grenada	AMR			125
Guatemala	AMR			17,608
Guinea	AFR			13,532
Guinea-Bissau	AFR			2061
Guyana	AMR			805
Haiti	AMR			11,448
Honduras	AMR			10,278
Hungary	EUR			9710
Iceland	EUR			370
India	SEAR			1,407,564
Indonesia	SEAR			273,753
Iran, Islamic Republic of	EMR			87,923
Iraq	EMR			43,534
Ireland	EUR			4987
Israel	EUR			8900
Italy	EUR			59,240
Jamaica	AMR			2828
Japan	WPR			124,613
Jordan	EMR			11,148
Kazakhstan	EUR			19,196
Kenya	AFR			53,006
Kiribati	WPR			129
Kuwait	EMR			4250
Kyrgyz Republic	EUR			6528
Lao People’s Democratic Republic	WPR			7425
Latvia	EUR			1874
Lebanon	EMR			5593
Lesotho	AFR			2281
Liberia	AFR			5193
Libya	EMR			6735
Lithuania	EUR			2787
Luxembourg	EUR			639
Madagascar	AFR			28,916
Malawi	AFR			19,890
Malaysia	WPR			33,574
Maldives	SEAR			521
Mali	AFR			21,905
Malta	EUR			527
Marshall Islands	WPR			42
Mauritania	AFR			4615
Mauritius	AFR			1299
Mexico	AMR			126,705
Micronesia, Federated States of	WPR			113
Monaco	EUR			37
Mongolia	WPR			3348
Montenegro	EUR			628
Morocco	EMR			37,077
Mozambique	AFR			32,077
Myanmar	SEAR			53,798
Namibia	AFR			2530
Nauru	WPR			13
Nepal	SEAR			30,035
Netherlands, The Kingdom of the	EUR			17,502
New Zealand	WPR			5130
Nicaragua	AMR			6851
Niger	AFR			25,253
Nigeria	AFR			213,401
Niue	WPR			2
North Macedonia	EUR			2103
Norway	EUR			5403
Oman	EMR			4520
Pakistan	EMR			231,402
Palau	WPR			18
Panama	AMR			4351
Papua New Guinea	WPR			9949
Paraguay	AMR			6704
Peru	AMR			33,715
Philippines	WPR			113,880
Poland	EUR			38,308
Portugal	EUR			10,290
Qatar	EMR			2688
Republic of Korea	WPR			51,830
Republic of Moldova	EUR			3062
Romania	EUR			19,329
Russian Federation	EUR			154,103
Rwanda	AFR			13,462
Saint Kitts and Nevis	AMR			48
Saint Lucia	AMR			180
Saint Vincent and the Grenadines	AMR			104
Samoa	WPR			219
San Marino	EUR			34
Sao Tome and Principe	AFR			223
Saudi Arabia	EMR			35,950
Senegal	AFR			16,877
Serbia	EUR			7297
Seychelles	AFR			106
Sierra Leone	AFR			8421
Singapore	WPR			5941
Slovakia	EUR			5448
Slovenia	EUR			2119
Solomon Islands	WPR			708
Somalia	EMR			17,066
South Africa	AFR			59,392
South Sudan	AFR			10,748
Spain	EUR			47,487
Sri Lanka	SEAR			21,773
Sudan	EMR			45,657
Suriname	AMR			613
Sweden	EUR			10,467
Switzerland	EUR			8691
Syrian Arab Republic	EMR			21,324
Tajikistan	EUR			9750
Tanzania, United Republic of	AFR			63,588
Thailand	SEAR			71,601
Timor-Leste	SEAR			1321
Togo	AFR			8645
Tonga	WPR			106
Trinidad and Tobago	AMR			1526
Tunisia	EMR			12,263
Türkiye	EUR			84,775
Turkmenistan	EUR			6342
Tuvalu	WPR			11
Uganda	AFR			45,854
Ukraine	EUR			43,531
United Arab Emirates	EMR			9365
United Kingdom of Great Britain and Northern Ireland	EUR			67,281
United States of America	AMR			336,998
Uruguay	AMR			3426
Uzbekistan	EUR			34,081
Vanuatu	WPR			319
Venezuela ^‡^	AMR			28,200
Vietnam	WPR			97,468
Yemen	EMR			32,982
Zambia	AFR			19,473
Zimbabwe	AFR			15,994
**Territory/Region**	**WHO Region**	Measles Elimination Category 2022 *	Rubella Elimination Category 2022 *	**Total, Population** **2021** **(Thousands), ^†^**
American Samoa (US)	WPR			45
French Polynesia (France)	WPR			304
Guam (US)	WPR			171
Hong Kong SAR (China)	WPR			7495
Macao SAR (China)	WPR			687
New Caledonia (France)	WPR			288
Northern Mariana Islands (US)	WPR			49
Occupied Palestinian Territories	EMR			5133
Pitcairn Islands (UK)	WPR			0,^§^
Tokelau (New Zealand)	WPR			2
Wallis and Futuna (France)	WPR			12
Category *	**Definition**			**Code**
Endemic	Continuous transmission of measles and/or rubella that persists for ≥12 months in any defined geographical area and no previous verification of elimination.			
Eliminated	Absence of endemic transmission for a continuous period of ≥12 months in the presence of a high-quality surveillance system.			
Verified	Verification of elimination for a region requires that all countries in the region document interruption of endemic virus transmission for a period of ≥36 months.			
Re-established endemic transmission post-verification	Presence of a chain of transmission that continues uninterrupted for ≥12 months in a defined geographical area (region or country) after previous verification of elimination.			
No report	National Verification Committee annual report not provided to the Regional Verification Commission for review			

Abbreviation: AFR = African Region; AMR = Region of Americas; EMR = Eastern Mediterranean Region; EUR = European Region, SAR = special administrative region SEAR = South-East Asia Region; UK = United Kingdom of Great Britain and Northern Ireland; US = United States of American, WPR = Western Pacific Region; WHO = World Health Organization. [* Categories for classifying the elimination status of countries and territories and definitions are derived from the Weekly Epidemiological Record (WER) 12 October 2018 (93): 544–552 [16]. Guidance for evaluating progress towards elimination of measles and rubella. https://iris.who.int/bitstream/handle/10665/275394/WER9341-544-552.pdf?sequence=1&isAllowed=y (accessed on 30 April 2024). ^†^ United Nations, Department of Economic and Social Affairs, Population Division (2022). World Population Prospect 2022, Online Edition. World Population Prospects—Population Division—United Nations. ^‡^ Re-verification of Venezuela at the Pan American Health Organization (PAHO)/World Health Organization (WHO) Region of the Americas, third annual meeting of the measles, rubella, and congenital rubella syndrome post-elimination Regional Monitoring and Re-Verification Commission held from 14–16 November 2023 and include a review of data from the first semester of 2023. ^§^ Only 50 permanent inhabitants—source: Government of The Pitcairn Islands http://www.government.pn/gpi-policies (accessed on 11 July 2024)].

## Data Availability

The data presented in this study are available in this article.
